# Mesenchymal Stem Cell-Derived Exosomes Ameliorated Diabetic Nephropathy by Autophagy Induction through the mTOR Signaling Pathway

**DOI:** 10.3390/cells7120226

**Published:** 2018-11-22

**Authors:** Nesrine Ebrahim, Inas A. Ahmed, Noha I. Hussien, Arigue A. Dessouky, Ayman Samir Farid, Amal M. Elshazly, Ola Mostafa, Walaa Bayoumie El Gazzar, Safwa M. Sorour, Yasmin Seleem, Ahmed M. Hussein, Dina Sabry

**Affiliations:** 1 Department of Histology and Cell Biology, Faculty of Medicine, Benha University, Benha 13518, QG, Egypt; nesrien.salem@fmed.bu.edu.eg (N.E.); ola.mostafa.moez@gmail.com (O.M.); 2 Stem Cell Unit, Faculty of Medicine, Benha University, Benha 13518, QG, Egypt; 3 Department of Medical Biochemistry, Faculty of Medicine, Benha University, Benha 13518, QG, Egypt; inas.ahmed@fmed.bu.edu.eg (I.A.A.); bioch_2004@yahoo.com (W.B.E.G.); 4 Molecular Biology and Biotechnology Unit, Faculty of Medicine, Benha University, Benha 13518, QG, Egypt; 5 Department of Physiology, Faculty of Medicine, Benha University, Benha 13518, QG, Egypt; drnohaibrahim79@gmail.com; 6 Department of Histology and Cell Biology, Faculty of Medicine, Zagazig University, Zagazig 44519, Egypt; arigueamir@yahoo.com; 7 Department of Clinical Pathology, Faculty of Veterinary Medicine, Benha University, Moshtohor, Toukh 13736, QG, Egypt; 8 Department of Anatomy, Faculty of Medicine, Benha University, Benha 13518, QG, Egypt; Amal.elshazly79@yahoo.com; 9 Department of Clinical Pharmacology, Faculty of Medicine, Benha University, Benha 13518, QG, Egypt; safwa.sorour@fmed.bu.edu.eg (S.M.S.); yasmeen.seleem@fmed.bu.edu.eg (Y.S.); 10 Department of Internal Medicine, Faculty of Medicine, Benha University, Benha 13518, QG, Egypt; drdabour@yahoo.com; 11 Department of Medical Biochemistry, Faculty of Medicine, Cairo University, Cairo 11562, Egypt; dinasabry@kasralainy.edu.eg; 12 Molecular Biology and Stem Cell Unit, Faculty of Medicine, Cairo University, Cairo 11562, Egypt

**Keywords:** diabetic nephropathy, exosomes, autophagy, mTOR

## Abstract

Background: Diabetic nephropathy (DN) is a serious complication of diabetes mellitus and a common cause of end-stage renal disease. Autophagy has a defensive role against kidney damage caused by hyperglycemia. Mesenchymal stem cell (MSC)-derived exosomes are currently considered as a new promising therapy for chronic renal injury. However, the renal-protective mechanism of exosomes on DN is not completely understood. We examined the potential role of MSC-derived exosomes for enhancement of autophagy activity and their effect on DN. In our study, we used five groups of rats: control; DN; DN treated with exosomes; DN treated with 3-methyladenine (3-MA) and chloroquine (inhibitors of autophagy); and DN treated with 3-methyladenine (3-MA), chloroquine, and exosome groups. We assessed renal function, morphology, and fibrosis. Moreover, ratios of the autophagy markers mechanistic target of rapamycin (mTOR), Beclin-1, light chain-3 (LC3-II), and LC3-II/LC3-I were detected. Additionally, electron microscopy was used for detection of autophagosomes. Results: Exosomes markedly improved renal function and showed histological restoration of renal tissues, with significant increase of LC3 and Beclin-1, and significant decrease of mTOR and fibrotic marker expression in renal tissue. All previous effects were partially abolished by the autophagy inhibitors chloroquine and 3-MA. Conclusion: We conclude that autophagy induction by exosomes could attenuate DN in a rat model of streptozotocin-induced diabetes mellitus.

## 1. Introduction

Diabetic nephropathy (DN) is a devastating complication of diabetes mellitus and a leading cause of end-stage renal disease (ESRD) worldwide [1]. According to statistics, nearly 347 million people worldwide have diabetes, and this number is expected to increase to 430 million by 2030. DN is becoming more prevalent and, to some extent, reaching epidemic proportions [1,2]. Various functional and structural changes are involved in the pathogenesis of DN, including hemodynamic changes, oxidative stress, mesangial cell expansion, glomerulosclerosis, and the development of fibrosis [3]. However, despite conventional therapies, such as those that ameliorate hyperglycemia and hypertension, effective therapeutic strategies to counteract and reverse the progression of DN are lacking [4,5,6].

Current therapeutic modalities for DN are directed at controlling the diabetes-associated metabolic and hemodynamic changes to slow disease progression, while no therapy exists to repair the imminent renal damage [7]. Nevertheless, over the past decade, mesenchymal stem cells (MSCs) have gained increasing interest as a novel regenerative therapy against renal damage [8]. MSCs have the ability to differentiate into a number of different lineages [9]. The therapeutic impact of applied MSCs largely relies on released factors, including exosomes [10]. Recently, cell-derived exosomes were demonstrated to be a unique mechanism of cell-to-cell communication [11]. Exosomes are cell-derived vesicles, 30–100 nm in diameter, and discharged in the microenvironment by several cell types, including stem cells and their progenitors [12]. Exosomes contain mRNAs, microRNAs (miRNA), and proteins that could be transferred to target cells inducing genetic and epigenetic changes in target cells [13]. In addition, horizontal transfer of vesicular mRNAs and miRNAs can lead to an angiogenic program in endothelial cells or regulate the phenotypes of injured cells [14].

Macroautophagy (hereafter referred to as autophagy) is an evolutionarily conserved homeostatic process that mainly plays a role in damaged organelle degradation and intracellular content digestion [15]. Autophagy is a tightly regulated process that eliminates cytotoxic protein aggregates and damaged organelles through lysosomal degradation and allows cells to recycle mitochondrial energy sources and maintain survival [15]. Impairment of autophagy in renal cells, particularly podocytes and tubular cells, has been implicated in the pathogenesis of various kidney diseases, including DN [16]. Therefore, improvement and restoration of autophagy are considered as promising therapeutic targets for DN [17].

Impairment of autophagy is implicated in the pathogenesis of DN via activation of the mechanistic target of rapamycin (mTOR) pathway [18]. mTOR exists as two separate signaling complexes, mTOR complex 1 (mTORC1) and mTORC2, which are regulating autophagic activity. In general, mTORC1 inhibits autophagy via phosphorylation of ULK1. Nutrient starvation induces autophagy primarily through the inhibition of mTORC1 [19]. Enhanced mTORC1 activity is seen in human and experimental type 1 and type 2 DN [20,21,22]. Thus, in the present study, we evaluated the effects of MSC-derived exosomes in ameliorating histological alterations in DN in order to clarify their role in inducing autophagy by modulating the mTOR signaling pathway.

## 2. Materials and Methods

### 2.1. Experimental Animals

Inbred adult male albino rats (250–270 g), eight weeks of age, were obtained from the Experimental Animal Unit, Faculty of Veterinary Medicine, Benha University, Egypt. All animals were housed in clean cages and given a standard diet and clean water ad libitum. Their environment was controlled in terms of light (12 h cycle starting at 8:00 AM) and room temperature (23 ± 3 °C). This study was carried out in strict accordance with the recommendations in the Guide for the Care and Use of Laboratory Animals of the National Institutes of Health (NIH publication 85–23, revised 2011). All protocols were approved by the institutional review board for animal experiments of the Faculty of Medicine, Benha University, Egypt.

### 2.2. Preparation of MSC-Derived Exosomes

MSC-derived exosomes were obtained from the supernatant of MSCs, representing conditioned media. First, rat bone marrow-derived MSCs (BM-MSCs) were prepared in the Central Lab, Faculty of Medicine, Benha University [23]. The MSCs were cultured in Dulbecco's Modified Eagle Medium (DMEM) without fetal bovine serum (FBS), but with 0.5% human serum albumin (HSA) (Sigma-Aldrich, St. Louis, MO, USA), overnight. The viability of the cells cultured overnight was more than 99%, as detected by trypan blue exclusion. Cells were plated at 4000 cells/cm^2^ for 7 days. On day 7, cells were trypsinized, counted, and replated in expansion medium at a density of 2000 cells/cm^2^ for another seven days (end of passage 1). The expansion was performed until the third passage.

The conditioned medium was collected and stored at –80 °C. The medium was centrifuged at 2000*g* for 20 min to remove debris, and then ultracentrifuged at 100,000× *g* in a SW41 swing rotor (Beckman Coulter, Fullerton, CA, USA) for 1 hour at 4 °C. Exosomes were washed once with serum-free M199 (Sigma-Aldrich) containing 25 mM 4-(2-hydroxyethyl)-1-piperazineethanesulfonic acid (HEPES) (pH = 7.4), and submitted to a second ultracentrifugation in the same conditions. Exosomes were labeled with PKH26 fluorescent linker dye to trace them in vivo. Exosomes were stored at –80 °C for the experiment.

### 2.3. Characterization of MSC-Derived Exosomes

#### Exosome Labeling with PKH-26

Exosomes were isolated from the supernatant of the first, second, and third passages of MSCs cultured in α-MEM deprived of FBS. The MSC-derived exosomes were fixed with 2.5% glutaraldehyde in HSA for 2 h. After they were washed, exosomes were ultracentrifuged and suspended in 100 µL HSA. A total of 20 µL of exosomes was loaded onto a formvar/carbon-coated grid, negatively stained with 3% aqueous phosphor-tungstic acid for 1 min, and observed by transmission electron microscopy (Hitachi H-7650, Hitachi, Tokyo, Japan) [24]. The protein content of the exosome pellet was quantified by the Bradford method (BioRad, Hercules, CA, USA) [24]. The dose of injected exosomes was adjusted to 100 µg protein/suspended in 0.2 ml PBS [25]. Additionally, PKH26 (Sigma-Aldrich, St. Louis, MO, USA) was used to confirm the exosome localization within the renal tissue. The exosome pellet was diluted with PKH-26 kit solution to 1 mL, and 2 μL of fluorochrome was added to this suspension and incubated at 38.5 °C for 15 min. After that, 7 mL of serum-free HG-DMEM was added to the suspension, then it was ultracentrifuged for second time at 100,000× *g* for 1 h at 4 °C. The final pellet was resuspended rapidly in HG-DMEM and stored at −80 °C for future injection in experimentally induced rats [26].

### 2.4. Western Blot for Characterization of Exosomes

The antibody used was antigen affinity-purified polyclonal sheep IgG anti-rabbit CD81 (Biolegend, Cat No.: 0349509) and CD63 (Biolegend, cat no.: 0353007). Protein was isolated from isolated exosomes using RIPA buffer. A total of 20 ng of protein was loaded and separated by SDS-PAGE on 4–20% polyacrylamide gradient gels. After incubation in 5% nonfat dry milk, Tris-HCL, and 0.1% Tween 20 for 1 h, primary antibodies (CD81 and CD63 polyclonal antibodies, and β-actin antibody) were added to one of the membranes containing specimen samples and incubated at 4 °C overnight with β-actin as a loading control. After washing the membrane twice with 1× TBST, appropriate secondary antibodies were then added and incubated for 2 h at room temperature. After being washed twice with 1× TBST, densitometric analysis of the immunoblots was performed to quantify the amounts of CD81, CD63 and β-actin against control sample by total protein normalization using image analysis software on the ChemiDoc MP imaging system (version 3) produced by BioRad (Hercules, CA, USA).

### 2.5. Experimental Chemicals

Streptozotocin powder was obtained from Sigma-Aldrich Chemical Co. (St. Louis, MO, USA). The powder was stored at –20 °C, and the amount needed was dissolved in 0.1 mol/L citrate buffer, pH 4.5, immediately before use.

3-Methyladenine was purchased from Sigma-Aldrich in powder form. The powder was dissolved in distilled water, and then placed in 50 °C bath water.

Chloroquine diphosphate salt was purchased from Sigma-Aldrich in powder form. The powder was dissolved in distilled water.

### 2.6. Induction of DN

Type I diabetes was induced in overnight fasted rats by a single intraperitoneal (IP) injection of freshly prepared STZ (60  mg/kg, dissolved in 0.1 M cold citrate buffer, pH 4.5). After STZ injection, rats acquired drinking water containing sucrose (15 g/L) for 48 h, to lessen the early death due to insulin discharge from partially injured pancreatic islets. Seventy-two hours later, rats were checked for hyperglycemia, and those with fasting blood sugar more than 250 mg/dL were included in the study. Diabetic rats received long-acting insulin (2–4 U/rat) via subcutaneous injection to maintain blood glucose levels in a desirable range (350 mg/dL) and to avoid subsequent development of ketonuria [27].

### 2.7. Experimental Design and Treatment Protocol

**Figure 1:**
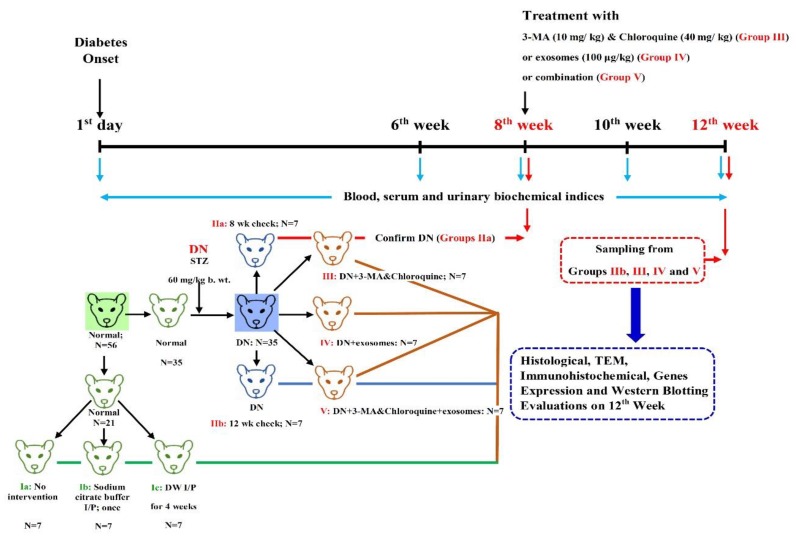
Experimental design and treatment procedure. Rats were divided into five groups: Group I (control group; *n* = 21 subdivided into three subgroups, seven rats each), Group II (DN group; *n* = 14 subdivided into two groups, seven rats each), Group III (DN + 3-MA and chloroquine; *n* = 7), Group IV (DN + exosomes; *n* = 7), and Group V (DN + 3-MA and chloroquine + exosomes; *n* = 7). Rats of DN groups were injected with streptozotocin (STZ) without/with treatment by 3-MA and chloroquine, exosome, or both. On the first day, and at weeks 6, 8, 10, and 12, blood samples were collected from all rats for biochemical assays, and at weeks 8 and 12, and renal tissues were collected for histological and molecular biological examinations.

The experimental design is shown in Figure 1. Fifty-six male rats were randomly divided into five groups as follows:

Group I (control group; *n* = 21): Rats were fed a regular chow diet for 12 weeks. The rats were divided equally into three subgroups of 7 rats each: 

Subgroup IA: The rats were left without intervention.

Subgroup IB: The rats were injected intraperitoneally with a single dose of 0.25 mL/kg body weight sodium citrate buffer (vehicle for STZ).

Subgroup IC: The rats were injected intraperitoneally with distilled water for four weeks (vehicle for chloroquine diphosphate). Simultaneously, they were intravenously injected with 0.2 mL phosphate-buffered saline (PBS), one injection at the 8th week. and the other injection at the 10th week of the experiment (vehicle for exosomes).

Group II (DN group; *n* = 14): DN was induced and the rats were subdivided equally into two subgroups:

Group IIA: DN was induced and rats were scarified at the end of the 8th week of the experiment to confirm histological changes of DN.

Group IIB: DN was induced and rats were scarified at the end of the experiment at the 12th week.

Group III (DN + 3-MA and chloroquine; *n* = 7): DN was induced and rats were intraperitoneally injected with 3-MA (10 mg/kg) and chloroquine (40 mg/kg) [28], once per day for four weeks, from the 8th week until the end of the experiment.

Group IV (DN + exosomes; *n* = 7): DN was induced and rats were treated with two injections of exosomes (100 μg/kg/dose suspended in 0.2 ml PBS) through the tail tail vein [25]. once per day for four weeks, from the 8th week until the end of the experiment.

Group IV (DN + exosomes; *n* = 7): DN was induced and rats were treated with two injections of exosomes (100 μg/kg/dose suspended in 0.2 mL PBS) through the tail vein [25]. The first injection was at the 8th week of the experiment, and the second at the end of the 10th week. 

Group V (DN + 3-MA and chloroquine + exosomes; *n* = 7): DN was induced and rats were intraperitoneally injected with 3-MA (10 mg/kg) and chloroquine (40 mg/kg), once per day for four weeks, from the 8th week until the end of the experiment. Simultaneously, exosomes were injected at the 8th and 10th weeks of the experiment.

### 2.8. Sampling

Blood samples were obtained from retro-orbital venous plexus on the first day, then the 6th, 8th, 10th, and 12th weeks, to measure blood glucose, serum creatinine, and blood urea nitrogen (BUN). Serum levels of creatinine and BUN were determined using an auto-analyzer (Hitachi 912 Auto-Analyzer, Hitachi, Tokyo, Japan). Fasting blood glucose was estimated by the glucose oxidase-peroxidase method (GOD-POD kit from Biodiagnostic, Giza, Egypt). Additionally, rats were located in metabolic cages for collection of 24 h urine, to measure urinary proteins at the same intervals. Urinary protein excretion was determined using (Fortress Diagnostics Ltd., Antrim, UK).

Nephropathy was confirmed in rats at the end of the 6th week by significant increases in protein in the urine, serum creatinine, and BUN, when compared with controls. Rats having these significant values were enrolled in the study. 

At the end of the 12th week, the rats were anesthetized by sodium thiopental anesthesia (40 mg/kg IP) after 12 h of fasting. The rats were fixed on an operating table and blood samples were obtained from retro-orbital venous plexus using a fine-walled Pasteur pipette. Then, vascular perfusion fixation through the left ventricle with 1% glutaraldehyde was performed. The kidneys were collected from the rats of all groups for histological and immunohistochemical analysis, transmission electron microscopy, qPCR study, and Western blot

### 2.9. Histological Analysis

#### 2.9.1. Light Microscopy Study

The specimens were excised, and paraffin sections of 4–6 µm thickness were created and then mounted on glass slides for H and E and Masson's trichrome stains [29]. Immunohistochemistry staining was done for detection of transforming growth factor β (TGF-β). The primary monoclonal antibody used was rabbit monoclonal antibody to TGF-β (Lab Vision/NeoMarkers, Fremont, CA, USA). Positive reaction was detected as a brown color in the cytoplasm [30].

#### 2.9.2. Transmission Electron Microscopy Study 

Vascular perfusion fixation through the left ventricle with 1% glutaraldehyde was performed, then the kidneys were dissected and 1 mm^3^ kidney samples were taken in 0.1 M phosphate-buffered solution (PBS), pH 7.4, at 4 °C for 2 h, then washed three times with PBS (10 min each). Samples were post fixed in 1% osmic acid for 30 min, then washed three times with PBS (10 min each). Samples were dehydrated with an ascending series of ethyl alcohol (30, 50, 70, 90%, and absolute alcohol) for 30 min at each concentration. Samples were infiltrated with acetone for 1 h, and then embedded in Araldite 502 resin. The plastic molds were cut using a Leica UCT ultramicrotome, then stained with 1% toluidine blue. After examination of semithin sections, ultrathin sections (50–60 nm thick) were cut, stained with uranyl acetate, then counterstained with lead citrate, examined, and photographed using a JEOL-JEM-100 SX electron microscope (Japan), electron microscope unit, Tanta University [31].

### 2.10. Morphometric Study

The mean area percentage of collagen fiber deposition by Masson’s trichrome and of TGF-β expression were quantified in five images from five nonoverlapping fields from each rat of each group using Image-Pro Plus version 6.0 (Media Cybernetics Inc., Bethesda, MD, USA).

#### 2.10.1. Determination of the Expressions of *LC3*, *mTOR*, and *Beclin-1* Genes by qPCR 

##### Total RNA Extraction and Reverse Transcription

Total RNA was extracted from frozen kidney tissue samples by TRIzol method (Invitrogen, USA) using RNeasy Mini Kit (Qiagen, Germany) as previously described [32]. Samples were quantified using a NanoDrop One spectrophotometer (Thermo Fisher Scientific, USA). RNA (1 µg) was reverse transcribed using a T100 Thermal Cycler (BioRad, USA) and the Maxima First Strand cDNA Synthesis Kit (Thermo Fisher Scientific, USA), following the manufacturer’s guidelines [33].

##### *Quantitative Real-Time PCR* 

Real-time PCR was performed according to the manufacturer’s instructions, using Maxima SYBR Green/ROX qPCR Master Mix (Thermo Fisher, USA), by Step One Plus Real-Time PCR System (Life Technologies, USA) [34]. The primer sequences were as follows: *GAPDH* forward, TGATTCTACCCACGGCAAGTT; *GAPDH* reverse, TGATGGGTTTCCCATTGATGA; *LC3-II* forward, ACTGCCGCCCTAAAGGTTAC; *LC3-II* reverse, CGAGGTCCAACCCACAAAGA; Beclin-1 forward, CGGCTCCTATTCCATCAAAA; Beclin-1 reverse, AACTGTGAGGACAC CCAAGC [35]; *mTOR* forward, TTGAGGTTGCTATGACCAGAGAGAA; and *mTOR* reverse, TTACCAGAAAGGACACCAGCCAATG [34]. The mRNA expression of each sample was determined after correction by *GAPDH* expression. The relative expression was calculated using the 2^–ΔΔCT^ method. The results are expressed as the n-fold difference relative to the control group. Each sample was assayed three times.

### 2.11. Western Blot

The mTOR, LC3-I, LC3-II, S6K1, p62, fibronectin, TGF-β, and β-actin antibodies used were purchased from Abcam (anti-fibronectin antibody (ab23750), rabbit polyclonal antibody [36], anti-TGF beta 1 antibody (ab92486), rabbit polyclonal antibody [37], anti-LC3-I/II antibody (ABC929, Sigma-Aldrich), rabbit polyclonal antibody [38], anti-S6K1 antibody (ab9366), rabbit polyclonal antibody [39], anti-SQSTM1/p62 antibody (ab155686), rabbit polyclonal antibody [40] and anti-mTOR antibody (ab2732), rabbit polyclonal antibody [41]). The proteins of renal tissues were extracted by RIPA lysis buffer, which was delivered by Bio Basic Inc. (Markham, ON, Canada). Extracted proteins were separated by SDS-PAGE on 4–20% polyacrylamide gradient gels. After incubation in 5% nonfat dry milk, Tris-HCL, and 0.1% Tween 20 for 1 h, primary antibodies (fibronectin, TGF-β, mTOR, LC3 II polyclonal antibodies, and β-actin antibody) were added to one of the membranes containing specimen samples and incubated at 4 °C overnight with β-actin as a loading control. After washing the membrane twice with 1× TBST, appropriate secondary antibodies were then added and incubated for 2 h at room temperature. After being washed twice with 1× TBST, densitometric analysis of the immunoblots was performed to quantify the amounts of collagen-I and β-actin against control sample by total protein normalization using image analysis software on the ChemiDoc MP imaging system (version 3) produced by BioRad (Hercules, CA, USA).

A statistical analysis was performed using the statistical software package SPSS for Windows (Version 18.0; SPSS Inc., Chicago, IL, USA). Differences between groups were evaluated using a one-way ANOVA followed by a Duncan post-hoc test. For each test, all the data are expressed as the mean ± standard error of mean (SE), and a *p* value <0.05 was considered significant.

## 3. Results

### 3.1. Exosome Characterization

**Figure 2:**
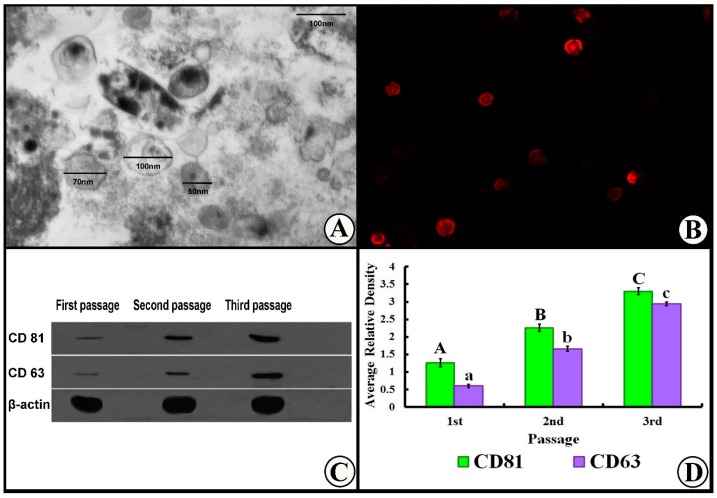
(**A**) TEM of exosomes showed spheroid double-membrane bound morphology (arrows) with a diameter of 40–100 nm. (**B**) Exosomes were also detected in renal tissues by PKH26. (**C**) Western blot for exosome characterization. (**D**) Histogram of exosome characterization. For CD81 panel, means with uppercase letters (A, B, and C) indicate significant differences at *p* ˂ 0.05. For CD63 panel, means with lowercase letters (a, b, and c) indicate significant differences at *p* ˂ 0.05. Data are shown as mean ± SEM, *n* = 5.

A transmission electron microscopy examination of purified exosomes demonstrated their characteristic spheroid double-membrane bound morphology with a diameter of 40–100 nm (Figure 2A). Also, the exosomes were detected in renal tissues by PKH26 dye tracing (Figure 2B). The amount of purified exosome was significantly (*p* ˂ 0.05) increased over the passages, and reached maximum concentration in the third passage, which was used for administration to rats (Figure 2 C,D).

### 3.2. Biochemical Analysis

**Figure 3:**
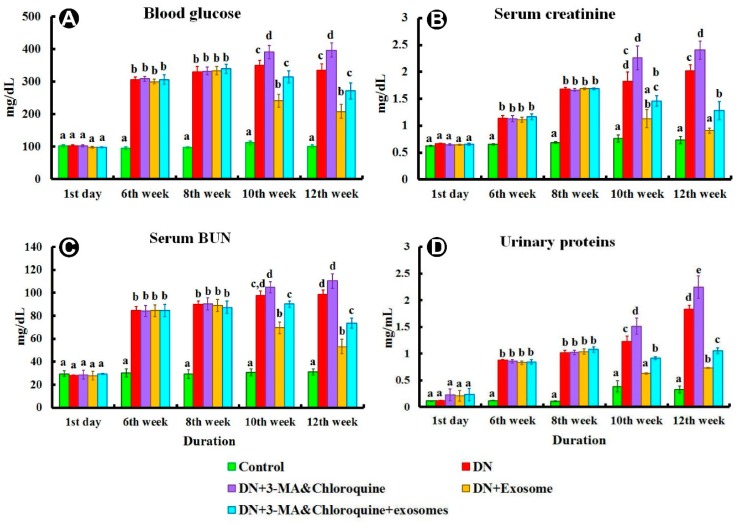
(**A**) Blood glucose, (**B**) serum creatinine, (**C**) blood urea nitrogen (BUN), and (**D**) urinary proteins from different experimental groups. Subgroup Ic was used as a control. Different superscripts (a, b, c, d, and e) at the same checkpoint indicate significant differences at *p* ˂ 0.05. Data are shown as mean ± SEM, *n* = 7.

Examination of all subgroups of the control group showed similar results regarding biochemical examinations; therefore, results of subgroup Ia were used to represent this group. Injection of streptozotocin (STZ) resulted in significant increases in blood glucose, serum creatinine, blood urea nitrogen (BUN), and urinary proteins excretion at the end of weeks 6, 8, 10, and 12, compared to the control group (*p* < 0.05) (Figure 3). Injection of exosomes caused significant decreases (*p* < 0.05) in blood glucose, serum creatinine, BUN, and urinary proteins excretion at weeks 10 and 12, compared with the DN group. On the other hand, treatment of DN with 3-MA and chloroquine aggravated the effects of injected STZ and reduced the protective effects of exosome, indicated by significant (*p* < 0.05) increases in blood glucose, serum creatinine, BUN, and urinary proteins excretion at weeks 10 and 12, when compared with the DN and exosome-treated groups. Similarly, the DN group treated with 3-MA and chloroquine and exosome showed significant (*p* < 0.05) increases in blood glucose, serum creatinine, BUN, and urinary proteins excretion at weeks 10 and 12 when compared with the exosome-treated group.

### 3.3. Histological Examination

Histological examination of the different subgroups of Group I (control group) showed similar results; therefore, results of subgroup Ia were used to represent this group.

#### 3.3.1. Light Microscope Examination

##### Hematoxylin and Eosin

**Figure 4:**
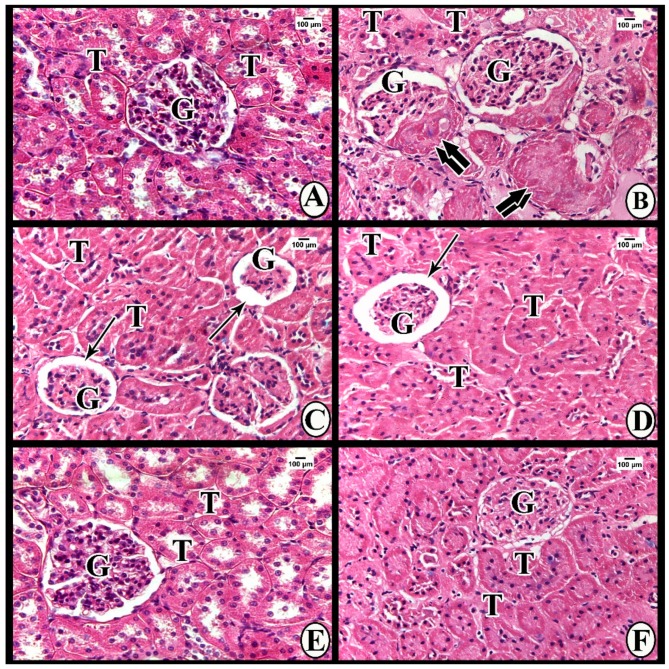
(**A**) Hematoxylin and eosin (H&E) stained sections of renal tissue of Group I show normal glomerulus (G) and tubules (T). (**B**) Group IIa shows mesangial expansion in the glomeruli (G) and glomerular nodular sclerosis (double arrows). Tubules (T) show obliterated lumens and darkly stained nuclei with loss of nuclear polarity. (**C, D**) Groups IIb and III demonstrate shrunken glomeruli (G) with wide Bowman’s spaces (thin arrows). The tubules (T) show swollen epithelial lining obliterating the lumens in addition to darkly staining nuclei with loss of polarity. (**E**) Group IV shows decreased mesangial expansion in the glomeruli (G). Tubules (T) have open lumens and normal nuclear polarity. (**F**) Group V shows persistent mesangial expansion in the glomeruli (G), swollen tubular epithelia (T) obliterating the lumens, and darkly stained nuclei with loss of polarity.

Sections of Group I (control group) revealed renal corpuscles consisting of glomeruli surrounded by narrow Bowman’s space and Bowman’s capsule. The corpuscles were surrounded by proximal and distal convoluted tubules (Figure 4A). Group IIa (DN group at the end of week 8): The renal tissues showed numerous glomeruli with mesangial expansion and glomerular nodular sclerosis (Kimmelstien–Wilson nodules), which appeared as acidophilic nodules with palisading nuclei at the periphery of glomeruli. ­­­Many tubules demonstrated darkly stained nuclei (Figure 4B). Also, sections of Group IIb (DN group at the end of week 12) demonstrated shrunken glomeruli with wide Bowman’s spaces (glomerulosclerosis). The tubules showed swollen epithelial lining obliterating their lumens, in addition to darkly staining nuclei. Some nuclei were basal, while others were apical (Figure 4C). The renal corpuscles of Group III (DN + 3-MA and chloroquine) showed marked shrinkage of glomeruli with obvious widening of the Bowman’s spaces (glomerulosclerosis). The tubules showed swollen tubular epithelium obliterating the lumen and darkly stained nuclei with loss of their polarity (some nuclei were basal, while others were apical) (Figure 4D). Glomeruli of Group IV (DN + exosomes) exhibited decreased mesangial expansion. The tubules showed open lumens and normal nuclear polarity (basal nuclei) of their lining epithelium (Figure 4E). Group V (DN + 3-MA and chloroquine + exosomes) demonstrated persistent mesangial expansion with swollen tubular epithelial lining, obliterated lumens, and darkly staining nuclei with loss of nuclear polarity (Figure 4F).

##### Masson’s Trichrome Stain

**Figure 5:**
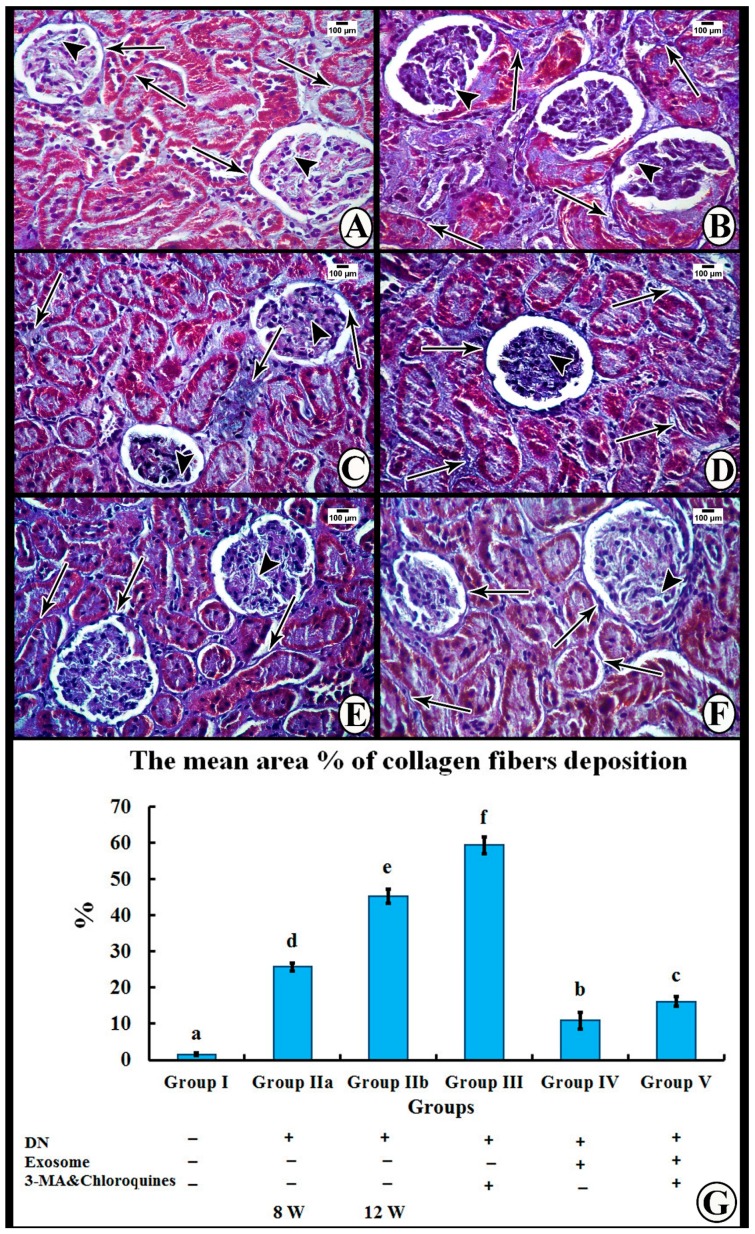
(**A**) Masson’s trichrome stained sections of renal cortex of Group I show minimal collagen fibers among the glomerular capillaries (arrowhead) and surrounding the renal corpuscles and tubules (thin arrows). (**B**) Group IIa demonstrates accumulation of collagen fibers among the glomerular capillaries (arrowheads) and surrounding the renal corpuscles and tubules (thin arrows). (**C**) Group IIb shows marked accumulations of collagen fibers among the glomerular capillaries (arrowheads) and surrounding the renal corpuscles and tubules (thin arrows). (**D**) Group III shows extensive accumulation of collagen fibers among the glomerular capillaries (arrowhead) and surrounding the renal corpuscles and tubules (thin arrows). (**E**) Group IV has few collagen fibers among the glomerular capillaries (arrowhead) and around the renal corpuscles and tubules (thin arrows). (**F**) Group V demonstrates persistent accumulation of collagen fibers among the glomerular capillaries (arrowhead) and surrounding the renal corpuscles and tubules (thin arrows). (**G**) A histogram represents the mean area percentage of collagen fiber deposition in all experimental groups. Different superscripts (a, b, c, d, e, and f) indicate significant differences among the experimental groups at *p* ˂ 0.05. Data are shown as mean ± SEM, *n* = 6.

Masson’s trichrome stained sections of Group I (control group) demonstrated minimal amounts of collagen fibers among the glomerular capillaries and surrounding the renal corpuscles and tubules (Figure 5A). Groups IIa and IIb (DN groups) and Group III (DN + 3-MA and chloroquine) showed an obvious increase in the amount of collagen fiber deposition among the glomerular capillaries and between the tubules, with a greater increase observed in Group III (Figure 5B–D). On the other hand, Group IV (DN + exosomes) revealed decreased amounts of collagen fibers among the glomerular capillaries and surrounding the renal corpuscles and tubules (Figure 5E), while Group V (DN + 3-MA and chloroquine + exosomes) demonstrated a persistent increase in collagen fibers among the glomerular capillaries and surrounding the renal corpuscles and tubules (Figure 5F).

### 3.4. Immunohistochemical Study

**Figure 6:**
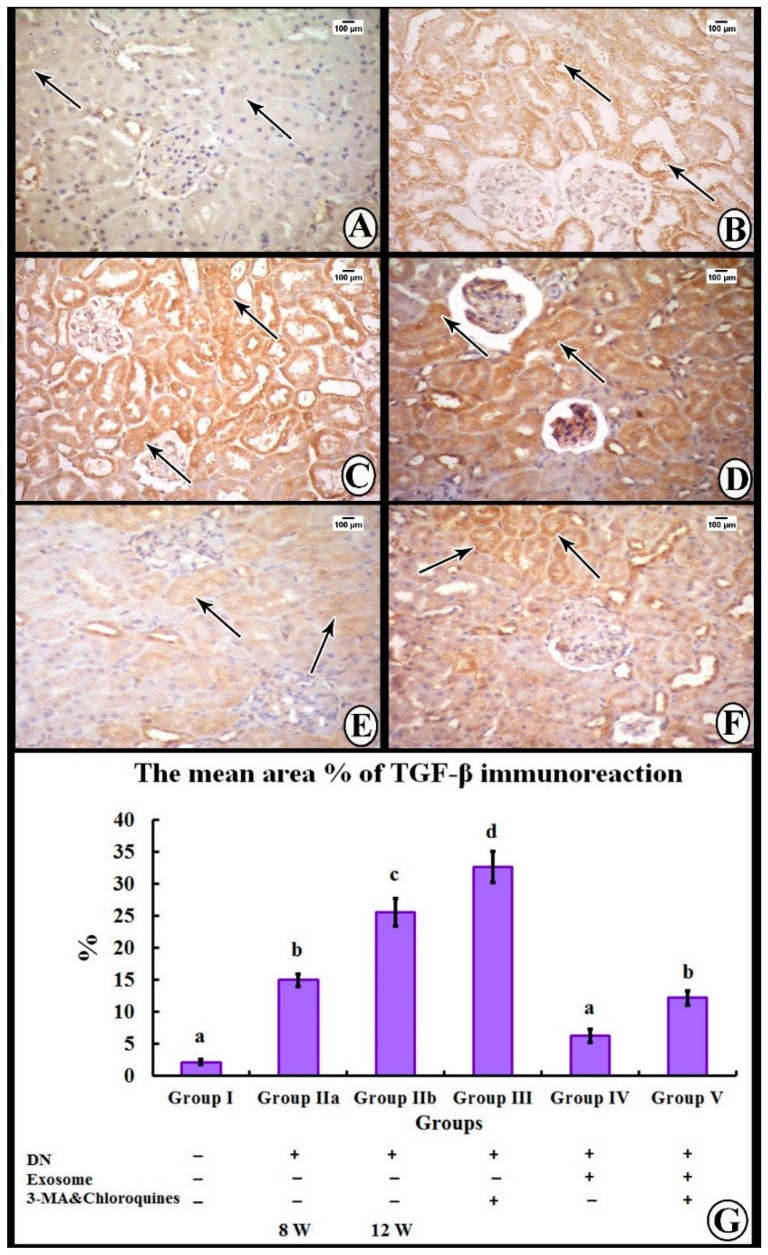
Immunohistochemical reaction for transforming growth factor β (TGF-β) in sections of the renal cortex. (**A**) Group I shows mild positive cytoplasmic reaction in the tubular epithelial cells (thin arrows). (**B**) Group IIa shows tubular cells with a moderate positive cytoplasmic reaction (thin arrows). (**C**) Group IIb shows a strong positive reaction in the cytoplasm of the tubular cells (thin arrows). (**D**) Group III shows intense positive reactions in the cytoplasm of the tubular cells (thin arrows). (**E**) Group IV shows a mild positive cytoplasmic reaction (thin arrows). (**F**) Group V tubular cells show a moderate positive cytoplasmic reaction (thin arrows). (**G**) Histogram representing the mean area percentage of TGF-β immunoreaction in all experimental groups. Different superscripts (a, b, c, and d) indicate significant differences among the experimental groups at *p* ˂ 0.05. Data are shown as mean ± SEM, *n* = 6.

The immunohistochemical reaction for transforming growth factor β (TGF-β) in Group I showed a minimal positive cytoplasmic reaction in the tubular epithelial cells (Figure 6A). In Group IIa, the tubular cells showed a moderate positive cytoplasmic reaction (Figure 6B), while the reaction was strongly positive in tubular cells of Group IIb (Figure 6C). Tubular epithelial cells of Group III showed an intensely positive cytoplasmic reaction (Figure 6D). On the other hand, Group IV revealed a mildly positive cytoplasmic reaction (Figure 6E), while Group V showed a moderately positive cytoplasmic reaction (Figure 6F).

### 3.5. Morphometric Analysis

The mean area percentage of collagen fiber deposition and TGF-β immunoexpression for all groups is presented in Figure 6G and Figure 4G. The diabetic nephropathy groups (Group IIa and IIb) and Group III showed a significant increase in the mean area percentage of collagen fiber deposition and TGF-β immunoexpression compared to the control group. Furthermore, administration of exosomes caused a significant decrease in the mean area percentage of collagen fiber deposition and TGF-β immunoexpression compared to both DN groups. On the contrary, administration of 3-MA and chloroquine caused a significant decrease in the antifibrotic effect of exosomes, as evidenced by a significant increase in the mean area percentage of collagen fiber deposition and TGF-β immunoexpression compared to the exosome-treated group.

### 3.6. Transmission Electron Microscopy Study

**Figure 7:**
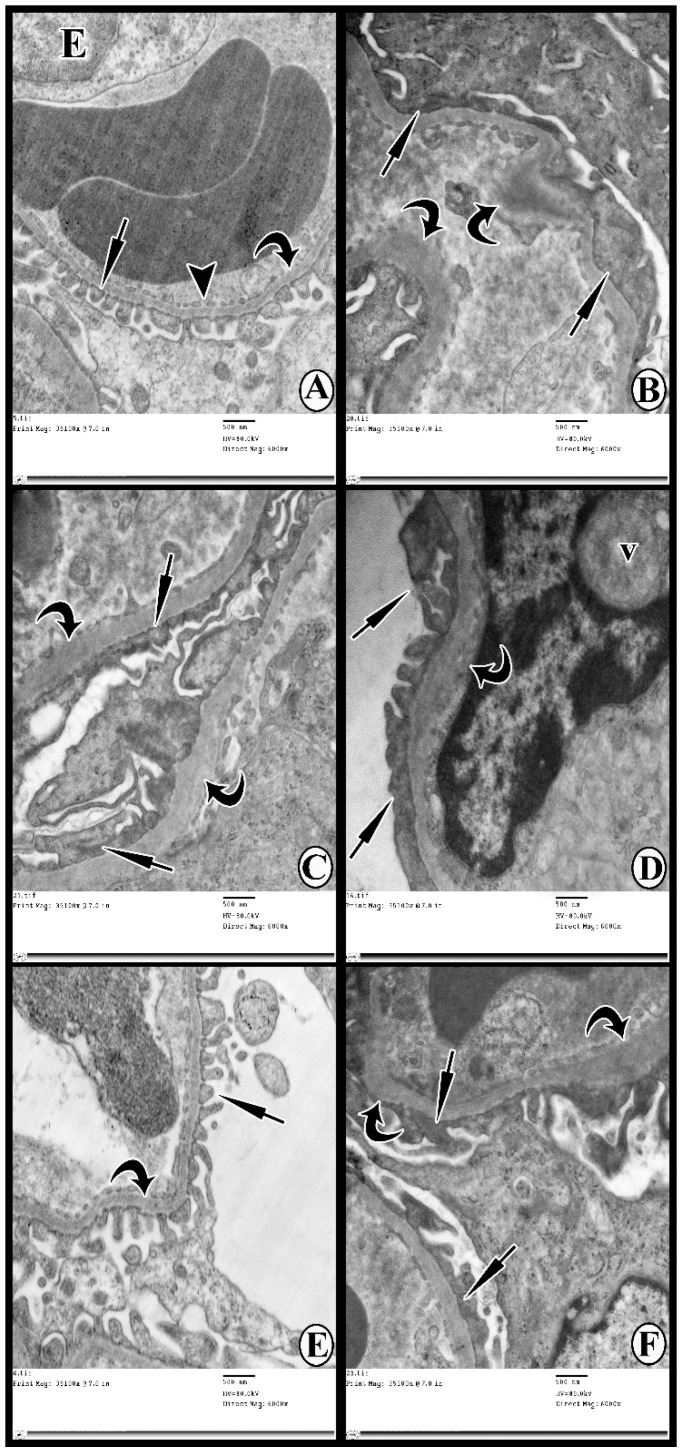
Ultrathin sections showing glomerular filtration barriers of (**A**) Group I, consisting of thin fenestrated endothelial cell (E; arrowhead), thin regular glomerular basement membrane (curved arrow), and the foot processes of the podocytes (short arrow). (**B**) Group IIa shows extensive fusion and effacement of the foot processes (short arrows) and diffuse thickening of the glomerular basement membrane (curved arrows). (**C**) Group IIb shows complete fusion and effacement of the foot processes (short arrows) and thickened glomerular basement membrane (curved arrows). (**D**) Group III shows areas of fusion and effacement of the podocytes (thin arrows) and diffuse thickening of the glomerular basement membrane (curved arrow). A vacuole (v) is seen compressing the nucleus of the endothelial cell. (**E**) Group IV shows thin regular glomerular basement membrane (curved arrow) and foot processes of podocytes (thin arrow). (**F**) Group V shows persistent fusion and effacement of the foot processes (short arrows) and thickening of the glomerular basement membrane (curved arrows) in some areas.

The ultrastructure of glomerular filtration barriers of Group I (control group) consisted of thin fenestrated endothelial cells and thin regular glomerular basement membranes, in addition to the foot processes of the podocytes (Figure 7A). Groups IIa and IIb (DN groups) demonstrated extensive fusion and effacement of the foot processes in addition to diffuse thickening of the glomerular basement membrane (Figure 7B,C). Group III (DN + 3-MA and chloroquine) showed that glomerular basement membranes had extensive areas of irregular thickening with areas of thickening, fusion, and effacement of the foot processes (Figure 7D). Group IV (DN + exosomes) revealed thin regular glomerular basement membranes in addition to the foot processes of the podocytes (Figure 7E). On the other hand, Group V (DN + 3-MA and chloroquine + exosomes) revealed persistent thickening of the glomerular basement membranes, and fusion and effacement of podocytes in many areas (Figure 7F).

**Figure 8:**
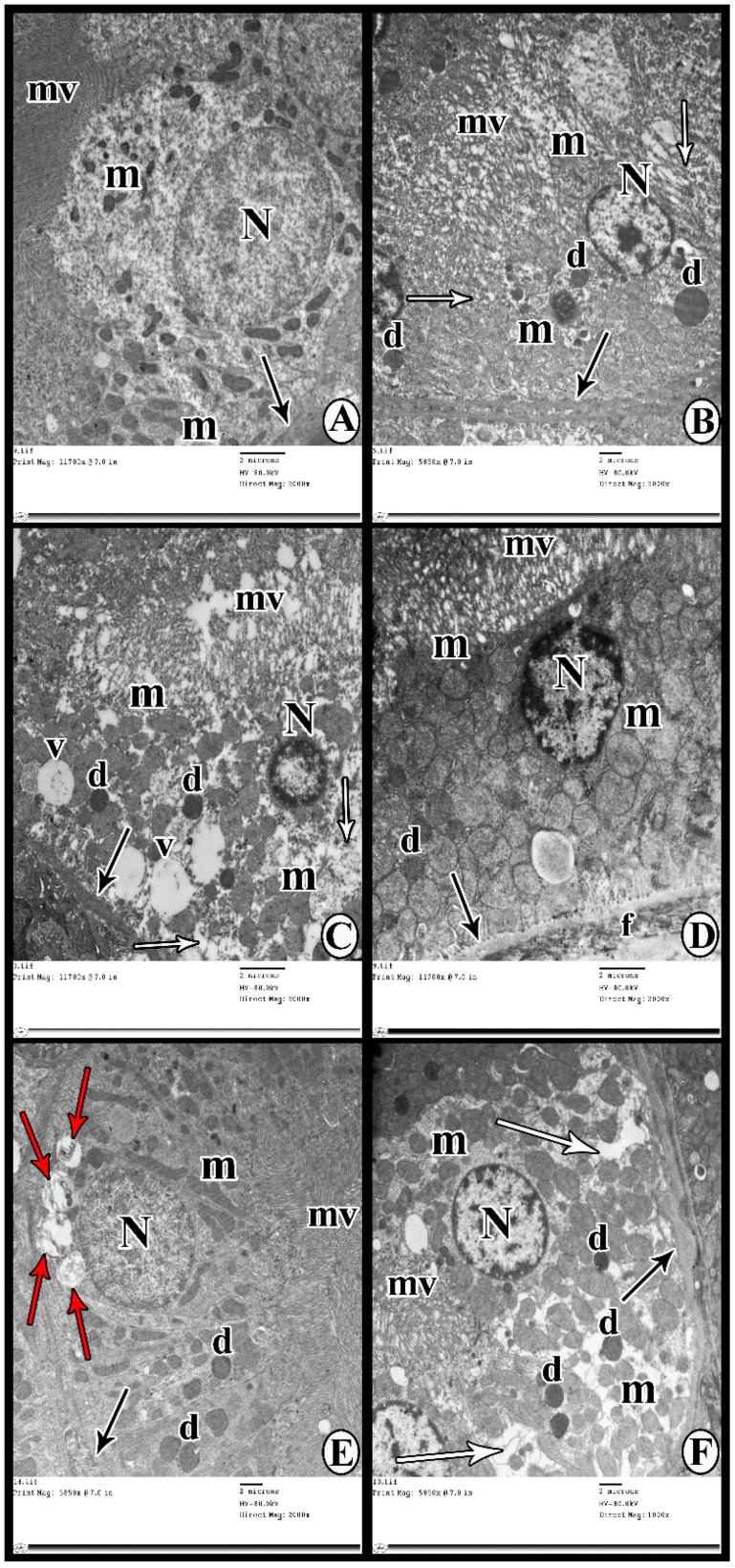
Ultrathin sections showing proximal tubular cells of (**A**) Group I resting on a thin regular basement membrane (thin arrow). Apical microvilli (mv) are numerous and tightly packed. The nucleus (N) appears oval and euchromatic, and the cytoplasm contains ­­numerous mitochondria (m). (**B**) Group IIa shows a slightly thickened tubular basement membrane (thin arrow), swollen disrupted apical microvilli (mv), and a shrunken nucleus (N). The cytoplasm shows areas of rarefaction (white arrows), numerous swollen mitochondria (m), and scattered electron-dense bodies (d). (**C**) Group IIb shows increased thickening of the tubular basement membrane (thin arrow), disrupted apical microvilli (mv), and a shrunken heterochromatic nucleus (N). The cytoplasm shows multiple variably sized vacuoles (v), variably sized mitochondria (m), and few scattered electron-dense bodies (d). (**D**) Group III shows a tubular cell resting on thickened basement membrane (thin arrow). Apical microvilli (mv) appear swollen and distorted. The nucleus (N) shows clumps of heterochromatin. The cytoplasm contains numerous swollen mitochondria (m) of various shapes and sizes. A small electron-dense body (d) is also observed. Thin collagen fibrils (f) are seen in the interstitium. (**E**) Group IV shows proximal tubular cells resting on a thin basement membrane (thin arrow). The apical microvilli (mv) appear numerous and tightly packed, and the nucleus (N) is round and euchromatic. The cytoplasm contains numerous autophagosomes (red arrows) containing cellular debris, numerous scattered electron-dense bodies (d), and elongated mitochondria (m). (**F**) Group V shows persistent thickening of the tubular basement membrane in some areas (thin arrow), swollen apical microvilli (mv), and slightly shrunken nucleus (N). The cytoplasm shows areas of rarefication (white arrows), variably sized mitochondria (m), and scattered electron-dense bodies (d).

The proximal tubular epithelial cells of Group I (control group) were seen resting on thin regular basement membranes. They possessed numerous tightly packed apical microvilli, round euchromatic nuclei, and numerous mitochondria in the cytoplasm (Figure 8A). Tubular cells of Group IIa (DN group after 8 weeks) rested on slightly thickened basement membranes. Their apical microvilli appeared swollen and disrupted, and the nuclei were shrunken. The cytoplasm contained areas of rarefaction in addition to numerous swollen mitochondria and scattered dense bodies (Figure 8B). Group IIb (DN group after 12 weeks) revealed increased thickening of the tubular basement membranes with disrupted apical microvilli and pyknotic nuclei. The cytoplasm contained multiple variably sized vacuoles, variably sized mitochondria, and few scattered electron-dense bodies (Figure 8C). Group III (DN + 3-MA and chloroquine) showed tubular epithelial cells resting on a thickened basement membrane with swollen and disrupted apical microvilli. The cytoplasm contained numerous swollen mitochondria of various sizes and shapes with few electron-dense bodies. The nuclei contained peripheral clumps of heterochromatin. Also, the interstitium revealed abundant collagen fibrils (Figure 8D). Group IV (DN + exosomes) showed the tubular cells resting on thin basement membranes. The apical microvilli were numerous and tightly packed, and the nuclei appeared round and euchromatic. The cytoplasm contained numerous autophagosomes containing cellular debris, numerous scattered electron-dense bodies, and elongated mitochondria (Figure 8E). In Group V (DN + 3-MA and chloroquine + exosome*s*), tubular cells showed persistent thickening of the tubular basement membranes in many areas, swollen apical microvilli, and slightly shrunken nuclei. The cytoplasm showed areas of rarefication in addition to variably sized mitochondria and scattered electron-dense bodies (Figure 8F).

### 3.7. Gene Expression Results of LC3-II, mTOR, and Beclin-1 Genes in All Experimental Groups

**Figure 9:**
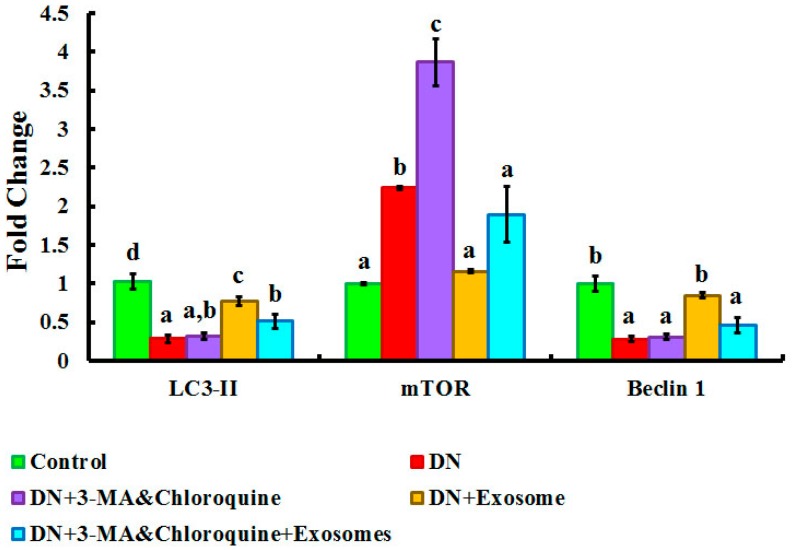
Quantitative analysis of relative light chain-3 (LC3-II), mechanistic target of rapamycin (mTOR), and Beclin-1 gene expression after treatment with 3-methyladenine (3-MA) and chloroquine and/or exosomes. Subgroup Ic was used as a control. Different superscript letters (a, b, and c) within each parameter panel indicate significant differences at *p* ˂ 0.05. Data are shown as mean ± SEM, *n* = 5.

Gene expressions of LC3-II, mTOR, and Beclin-1 genes are presented in Figure 9. The DN group showed significant (*p* < 0.05) downregulation of LC3 and Beclin-1 with significant (*p* < 0.05) upregulation in mTOR gene expression in renal tissues compared to the control group. Furthermore, treatment of DN with exosomes caused significant (*p* < 0.05) upregulation of Beclin-1 and LC3-II, as well as significant (*p* < 0.05) downregulation of mTOR gene expression in renal tissues when compared with the DN group. On the other hand, injection of both 3-MA and chloroquine caused significant (*p* < 0.05) downregulation of autophagy markers Beclin-1 and LC3-II, with significant (*p* < 0.05) upregulation of mTOR mRNA in renal tissues compared to the exosome-treated group.

### 3.8. mTOR, LC3-I, LC3-II, S6K1, p62, Fibronectin, TGF-β, and β-Actin Detection by Western Blotting

**Figure 10:**
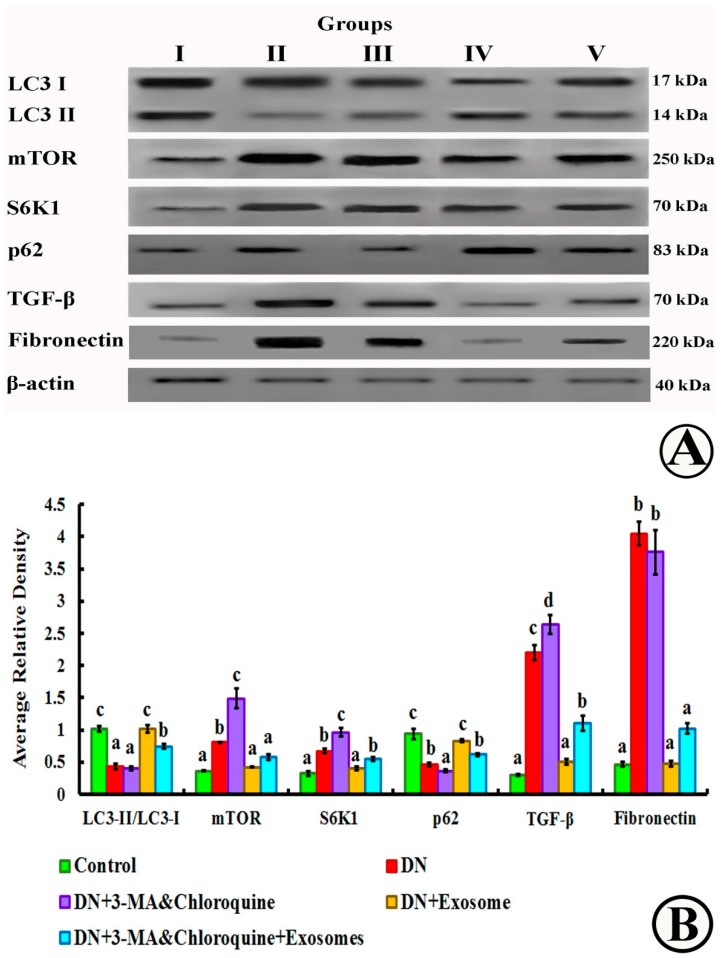
(**A**) Western blot assay and downstream target proteins for mTOR, LC3-I, LC3-II, S6K1, p62 fibronectin, TGF-β, and β-actin expression after treatment with 3-MA, chloroquine, and/or exosomes, and quantified using image analysis software on the ChemiDoc MP imaging system. (**B**) Histogram of blotting intensity in different groups Group I (control), Group II (DN), Group III (DN + 3-MA and chloroquine), Group IV (DN + exosomes), and Group V (DN + 3-MA and chloroquine + exosomes). Subgroup Ic was used as a control. Different superscript letters (a, b, c, and d) within each parameter panel indicate significant differences at *p* ˂ 0.05. Data are shown as mean ± SEM, *n* = 5

As shown in Figure 10, protein expressions of mTOR, fibronectin, S6K1 and TGF-β in the DN group were significantly (*p* < 0.05) higher than in the control group, and exosome treatment significantly (*p* < 0.05) reduced their levels. On the other hand, the ratio of LC3-II and LC3-I as well as p62 protein expression (Figure 10B) in the DN group decreased significantly when compared with the control group, while exosome treatment significantly enhanced LC3-II/LC3-I and p62 protein expression compared to the DN group (*p* < 0.05). Furthermore, injection of both 3-MA and chloroquine resulted in significant increases in fibronectin, TGF-β, S6K1, and mTOR protein expression, and a significant decrease in LC3-II and p62 protein expression compared with the exosome-treated group.

## 4. Discussion

The rapidly increasing prevalence of diabetes is resulting in a concomitant increase in the prevalence of DN [42]. Given the large worldwide healthcare burden associated with DN, there has been much interest in the search for novel treatment targets. Therefore, in recent decades, many researchers have been making a great deal of effort to recognize the molecular mechanisms in initiation and progression of diabetic nephropathy, in order to develop novel therapeutic approaches. However, end-stage kidney disease, due to DN, is still increasing worldwide. There is an urgent need to find additional novel therapeutic agents for the prevention of DN [43,44]. The present study demonstrates, for the first time, that MSC-derived exosomes could ameliorate DN by autophagy regulation through the mTOR signaling pathway in vivo in kidneys of diabetic rats.

The current study shows marked deterioration of renal function in DN rats, indicated by significant increases in serum creatinine, BUN, and urinary proteins excretion (Figure 3), supported by the histological lesions of classical DN, which were demonstrated by light (Figure 4, Figure 5 and Figure 6) and electron (Figure 7 and Figure 8) microscopy, in the form of thickening of the glomerular basement membrane and mesangial expansion, deformity of the filtration barrier, and interstitial fibrosis with diffuse glomerular sclerosis. These findings coincide with previous reports on patients with DN [45,46]. Glomerular cell loss is considered to be a result of diabetes-induced nephrotoxicity [47]. Furthermore, diabetes-induced nephrotoxicity affects the diameter of blood vessels; a vasodilatation effect [48]. Glomerular vasodilatation could induce podocyte mechanical stretch, leading to foot process effacement and, after that, cellular detachment. In agreement with what has been published [49,50], podocyte stretch induces decreased expression of podocyte nephrin, the main slit diaphragm protein, leading to disturbances in glomerular filtration function and proteinuria. Furthermore, accumulation of edematous fluids in diabetic patients induces an increase in Bowman's space. The two main causes of glomerular edema are increased movement of renal fluid from the glomerular pool to the urinary pool and blockade of the renal tubular system. As described before [51,52], dilated glomerular vessels could increase the vascular endothelium fenestrae, leading to increased fluid movement from the glomerular pool to the urinary pool, inducing edema formation. Additionally, Lenoir et al. [53] showed mesangial expansion and glomerulosclerosis by histological analysis and glomerular basement membrane thickening, podocyte foot process broadening, and effacement by TEM in endothelial cell-specific Atg5-deficient diabetic glomeruli.

Autophagy generated by various harmful factors contributes to the maintenance of cell homeostasis. Autophagy principally serves an adaptive or “programmed cell survival” mechanism to protect organisms during periods of enhanced cellular distress [54]. In our study, the deterioration of renal function in the DN group was possibly caused by defect(s) in the autophagy process. We evaluated LC3, Beclin-1, and mTOR, and conducted TEM examination of autophagosomes as proper procedures for monitoring autophagy [55]. The present study demonstrated significant downregulation of autophagy markers LC3II and Beclin-1 in renal tissue, as well as a significant upregulation of mTOR expression in the DN rats compared with the controls (Figure 9). These outcomes are in concurrence with those of Fang et al. [56], who found that the renal expression of autophagy-related proteins, including Beclin-1 and LC3II, was markedly suppressed in rats with DN. Also, the findings of the present study support the findings of Gödel et al. [22], who reported significant upregulation of mTOR itself and mTORC1 target genes in human patients with progressive DN, as compared to the controls. Gödel and colleagues also demonstrated that activation of mTORC1 inhibits diabetes-related autophagy in renal tissues of diabetic mice and patients. Nowadays, the gold standard for autophagy monitoring is TEM, the only technique able to elucidate the real presence of subcellular autophagic structures like autophagosome, lysosome, and autophagolysosome [55,57]. In the current study, TEM showed rare autophagic vacuoles in the tubular cells of the DN group compared to the control group (Figure 7 and Figure 8). In line with these results, Lenoir et al. [53] evaluated the role of autophagy in the endothelial cells of diabetic kidneys using endothelial cell-specific Atg5-deficient mice. Lenoir explained that the ultrastructural analysis exhibited glomerular endothelial cell cytoplasmic disorganization and vacuolization, as well as detached cells—most likely endothelial cells—in the lumen of the capillaries of the glomeruli of endothelial cell-specific Atg5-deficient diabetic mice. 

As a central element for signaling cell growth and enhancing protein translation, mammalian target of rapamycin (mTOR), when inhibited, induces autophagy. Likewise, as a critical feedback mechanism, reactivation of mTOR terminates autophagy and initiates lysosome reformation [54]. In our study, we found that rats with DN showed significant upregulation of mTOR mRNA, as well as protein expression. This upregulation is aggravated by 3-MA and chloroquine administration, and alleviated by exosome treatment (Figure 9 and Figure 10). Numerous studies have revealed that hyperactivation of the mTOR pathway in DN has an essential role in glomerular and tubular cell hypertrophy [58,59], and is related to injury of podocytes and decline of glomerular filtration rates. A growing number of studies have suggested that mTORC1 pathway inhibition with rapamycin has renoprotective effects on DN progression in models of type 1 [60] and type 2 [61] diabetes. Overactivation of mTOR resulted from prolonged exposure to hyperglycemia, which is a crucial factor in diabetic kidney injury [62,63]. These studies demonstrated that overactivation of mTOR in the podocytes results in albuminuria, due to the widening of glomerular basement membrane, mesangial expansion, and collagen deposition. Also, they showed that mTORC1 hyperactivity in podocytes disrupts localization of the proteins constructing the filtration slits, with eventual increased glomerular permeability to macromolecules. Additionally, other studies attributed progressive renal tissue injury to activation of mTORC1 and suppression of autophagy, and explained this issue by different mechanisms. First, defective autophagy impairs clearance of advanced glycation end products (AGEs) [64] and damages mitochondria in podocytes [17]. Second, autophagic insufficiency further increases hypoxia and endoplasmic reticulum (ER) stress, thus increasing the vulnerability of tubular cells [44,64]. Third, suppressed autophagy leads to collagen accumulation due to defective degradation [17].

More importantly, suppression of autophagy due to mTOR activation was studied by Fang et al. [56], who reported that autophagy initiation is triggered by the action of unc-51-like kinase 1 (ULK1) (mammalian ortholog of the yeast Atg1), which binds to and phosphorylates other autophagy essential proteins such as ribosome protein subunit 6 kinase 1 (S6K1) and eIF4E-binding protein 1 (4EBP-1), to inhibit autophagy through affecting the transcription and translation of related proteins [65]. However, exposure to hyperglycemia leads to activation of mTORC1 which, in turn, phosphorylates and inactivates ULK1, thus preventing initiation of autophagosome formation [66]. Of note, mTORC1 activation not only inhibits autophagy, but also is implicated in the synthesis of proteins and other macromolecules. It has been reported that activated mTOR enhances interstitial fibrosis in diabetic kidneys [20]. These findings are parallel to those of the present study, as we demonstrated a significant increase in TGF-β1 and fibronectin protein expression (Figure 10), and collagen fiber deposition among the glomerular capillaries and surrounding the renal corpuscles and tubules in renal specimens stained by Masson's trichrome in DN rats, as compared to controls (Figure 5). It has been suggested that hyperglycemia-activated mTORC1 stimulates fibroblast proliferation and collagen synthesis. Furthermore, it enhances the expression of profibrotic cytokines, such as TGF-β1 and connective tissue growth factor, resulting in progressive fibrosis and tubulointerstitial changes in DN [20]. Fibrosis considered as a noteworthy step of the pathogenesis of DN. Myofibroblasts assume an imperative job in the initiation of fibrosis in both the glomerulus and renal tubulointerstitium. Epithelial-to-mesenchymal transition (EMT) is a main source of myofibroblasts in podocytes and proximal tubular cells in DN. TGF-β has directly targeted several signaling pathways involving not only EMT induction, but also the synthesis of extracellular matrix molecules, such as fibronectin, collagen type I, and laminin, causing renal fibrosis. Therefore, regulation of TGF-β is a potential therapeutic target for DN cells [67]. 

The results of the present study also demonstrate that multiple injections of exosomes in Group IV significantly improved renal function in the DN group, indicated by significant decreases in serum creatinine, BUN, and urinary proteins (Figure 3). Moreover, renal histological changes improved in the form of decreased mesangial expansion and open-walled tubules (Figure 4), with a significant decrease in collagen expression by Masson’s trichrome staining and TGF-β immunoexpression (Figure 5). These results were supported by TEM, which demonstrated multiple autophagic vacuoles, euchromatic nuclei, scattered mitochondria, and closely packed apical microvilli, in proximal tubule epithelial cells (PTECs). Also, there were nearly normal glomerular basement membranes with nearly normal podocytes, and more thinning of the glomerular filtration barrier (Figure 6 and Figure 7).

These results were in accordance with Nassar et al. [68], who demonstrated that MSC administration could reverse DN through paracrine mechanisms, rather than by MSC transdifferentiation. MSC-derived exosomes might be such a paracrine mechanism for cell-to-cell communication. Evidence from previous studies indicate that MSCs mediate their paracrine effects via release of exosomes that deliver their cargo of various mRNAs, miRNAs, and proteins to recipient cells [23]. Additionally, Nagaishi et al. [67] proved that kidney that received exosome injection exhibited improvement in the histological picture of DN in the form of decreased degeneration, vacuolation, atrophic changes, and inflammatory cell infiltration of PTEC, confirmed by H and E staining, with decreased fibrous components, as confirmed by Azan staining.

The nephroprotective effect of exosomes was explained by Bruno et al. [23], who found that the exosomes got from MSCs exhibit defensive impacts in acute renal damage through the transportation of a particular subset of cell mRNAs, which are associated with the mesenchymal phenotype and with the control of transcription, proliferation, and immunoregulation. Furthermore, Bruno et al. revealed that exosomes initiated a proliferative program of PTEC and hindered apoptosis through inducing the synthesis of hepatocyte growth factor and macrophage-stimulating protein by the horizontal transfer of mRNAs packed in the exosomes. 

However, the accumulation of autophagic vacuoles did not always mean increased autophagosome formation and may denote inhibited autolysosome maturation, so LC3-II detection via Western blotting was valuable in monitoring the autophagosome number [44]; therefore, we detected the autophagosome formation-related proteins (LC3 conversion and Beclin-1). We also detected the autophagic flux by measuring the protein expression of p62 that serves as a connection between LC3 and ubiquitinated substrates [69]. We found that the DN group showed significant decreases in the expression level of LC3 and Beclin-1 compared with control. Additionally, accumulation of p62 protein has been observed DN group, indicating a deficiency in autophagy. Exosome-treated DN rats significantly increased the expression of Beclin-1 mRNA as well as the LC3-II/LC3-I and p62 protein expression ratio, indicating increased autophagosome formation (Figure 10). Accordingly, our results revealed that injections of exosomes upregulated the expression of the autophagy markers LC3 and Beclin-1, and downregulated mTOR and S6K1 in Group IV as compared to rats of the DN group, suggesting that exosomes may activate autophagy by decreasing mTOR. These results run parallel to an earlier study that reported that genetic reduction of mTOR levels in mTORC1 knockout mice significantly reduced the effects of mTOR hyperactivation in renal tissues and suppressed the progression of DN in diabetic animals [61]. As a limitation of our study, the expression of phosphorylated products of mTOR and S6K1 were not included during monitoring of autophagic activity in various study groups, however; therefore, it is recommended to be addressed in further studies.

In this context, our data were in accordance with earlier results of Liu and colleagues [70], who showed that MSC-derived exosomes significantly reduced the expression of phosphorylated mTOR/mTOR in rat cardiomyocytes after ischemia reperfusion injury. They attributed the enhanced autophagic activities in exosome-treated cells partly to inhibition of the mTOR pathway. These results provide a possible explanation for the improved biochemical and histological features of DN in Group IV.

Regarding the effects of exosomes on renal fibrosis induced by diabetes, repeated injection of MSC-derived exosomes in Group IV caused a significant decrease in the immunoexpression of TGF-β1 and collagen fiber deposition between glomeruli and tubules of renal samples stained by Masson’s trichrome, and these findings were confirmed by a significant decrease in protein expression of TGF-β and fibronectin by Western blot. Such results suggest that multiple injections of exosomes exhibit a potent antifibrotic effect and, therefore, may improve renal function indirectly by reducing disease-associated fibrosis. These outcomes run parallel to former reports [65]. suggesting that barricading the mTOR pathway decreased TGF-β1 in kidney. Previous studies showed that exosomes with selective microRNA patterns improved renal function and reversed TGF-β1 morphological changes in renal cells [67]. Thus, MSC-derived exosomes provide a new, potentially effective therapeutic approach to slow down the progression of renal disease and improve renal function, by modulating autophagy.

To confirm the role of autophagy in mediating the protective impact of exosomes on DN, we used both 3-MA and chloroquine, autophagy inhibitors, in Groups III and V. Our study demonstrated that both 3-MA and chloroquine (CQ) are beneficial in inhibiting the process of autophagy (Group III). 3-MA is an early autophagy inhibitor, which could suppress the activity of type III of phosphoinositide-3-kinase (PI3K) and the formation of autophagosome to inhibit autophagy [71], while chloroquine reverses autophagy by inhibiting lysosomal acidification, resulting in lysosome accumulation and autophagy blockade [28]. We used both 3-MA and chloroquine to be sure of autophagy inhibition, because a previous study revealed that, in nutrient-rich conditions such as ours, 3-MA may even stimulate autophagy flux instead of blocking it [72].

Treatment of DN rats with chloroquine and 3-MA (Group III) resulted in significant downregulation of LC3 and Beclin-1 (Figure 9). In addition, there was a reduction in the number of autophagic vacuoles, confirmed by TEM, when compared to the exosome-treated group (Figure 7 and Figure 8), indicating that autophagy was inhibited by 3-MA and chloroquine. Furthermore, pretreatment with chloroquine and 3-MA exacerbated renal insult, manifested by significant increases in serum creatinine, BUN, and urinary proteins (Figure 3), in parallel with deterioration of renal histology (Figure 4 and Figure 5). Such deterioration was evidenced by persistent mesangial expansion, swollen tubular epithelial lining with obliteration of their lumens, and darkly staining nuclei with loss of their polarity. Inhibition of autophagy by chloroquine not only decreased the protective effect of exosomes on renal function (Group V), but also decreased their antifibrotic effect, indicated by a significant increase in the protein expression of TGF-β (Figure 6) and fibronectin (Figure 10) with collagen fiber deposition (Figure 5), leading to persistent thickening of the glomerular basement membranes in many areas when compared to the exosome-treated group. In this context, our data were in accordance with earlier results of Verschooten and colleagues, who showed that chloroquine is an autophagy inhibitor enhancing the cell death-inducing effect of the flavonoid luteolin in metastatic squamous cell carcinoma cells. In our study, combined treatment of chloroquine and 3-MA with exosomes in Group V alleviated what was found in Group III [73].

## 5. Conclusions

In conclusion, the potential nephroprotective effects of MSC-derived exosomes in a diabetic nephropathy model are based on their ability to upregulate autophagy associated with suppression of mTOR pathway, and by their antifibrotic effect. These findings provide a basis for the future use of exosomes as a new biological therapeutic approach for DN.

## Authors Contributions

Conceptualization: N.E., I.A.A., N.I.H., A.A.D., and A.S.F.; methodology: A.S.F., N.E., I.A.A., N.I.H., A.A.D., and D.S.; validation: O.M. and W.B.E.G.; investigation: N.E., D.S., I.A.A., N.I.H., O.M., and A.S.F.; resources: N.E., A.A.D., O.M., A.M.H., N.I.H., A.M.E., W.B.E.G., S.M.S., and Y.S.; formal analysis: A.A.D., A.S.F., N.I.H., and N.E.; data curation: A.A.D., O.M., N.I.H., and A.S.F.; writing—original draft preparation: N.E., N.I.H., I.A.A., and A.S.F.; writing—review and editing: A.S.F., N.E., N.I.H,. I.A.A., and A.A.D.; supervision, paper writing, and final editing: A.S.F., A.A.D., N.E., Y.S., and S.M.S.

## Abbreviations

3-MA: 3-methyladenine; BUN: blood urea nitrogen; DN: diabetic nephropathy; ESRD: end-stage renal disease; LC3: Microtubule-associated protein 1A/1B-light chain 3; MSCs: mesenchymal stem cells; mTOR: mechanistic target of rapamycin; STZ: Streptozotocin; TGF: Transforming growth factor; ULK1: Unc-51 like autophagy activating kinase.

## References

[1] Martínez-Castelao A., Navarro-González J.F., Górriz J.L., de Alvaro F. (2015). The concept and the epidemiology of diabetic nephropathy have changed in recent years. J. Clin. Med..

[2] Saran R., Robinson B., Abbott K.C., Agodoa L.Y., Albertus P., Ayanian J., Balkrishnan R., Bragg-Gresham J., Cao J., Chen J.L. (2017). US renal data system 2016 annual data report: Epidemiology of kidney disease in the United States. Am. J. Kidney Dis..

[3] Forbes J.M., Coughlan M.T., Cooper M.E. (2008). Oxidative stress as a major culprit in kidney disease in diabetes. Diabetes.

[4] Lorenzen J.M., Haller H., Thum T. (2011). MicroRNAs as mediators and therapeutic targets in chronic kidney disease. Nat. Rev. Nephrol..

[5] Fernandez-Fernandez B., Ortiz A., Gomez-Guerrero C., Egido J. (2014). Therapeutic approaches to diabetic nephropathy—Beyond the RAS. Nat. Rev. Nephrol..

[6] Ahmad J. (2015). Management of diabetic nephropathy: Recent progress and future perspective. Diabetes Metab. Syndrome.

[7] Satirapoj B., Adler S.G. (2014). Comprehensive approach to diabetic nephropathy. Kidney Res. Clin. Pract..

[8] Tögel F., Weiss K., Yang Y., Hu Z., Zhang P., Westenfelder C. (2007). Vasculotropic, paracrine actions of infused mesenchymal stem cells are important to the recovery from acute kidney injury. Am. J. Physiol..

[9] Ullah I., Subbarao R.B., Rho G.J. (2015). Human mesenchymal stem cells-current trends and future prospective. Biosci. Rep..

[10] Katsuda T., Kosaka N., Takeshita F., Ochiya T. (2013). The therapeutic potential of mesenchymal stem cell-derived extracellular vesicles. Proteomics.

[11] Roy S., Hochberg F.H., Jones P.S. (2018). Extracellular vesicles: the growth as diagnostics and therapeutics; a survey. J. Extracell. Vesicles.

[12] Yeagy B.A., Cherqui S. (2011). Kidney repair and stem cells: a complex and controversial process. Pediatric Nephrol..

[13] Zarjou A., Kim J., Traylor A.M., Sanders P.W., Balla J., Agarwal A., Curtis L.M. (2011). Paracrine effects of mesenchymal stem cells in cisplatin-induced renal injury require heme oxygenase-1. Am. J. Physiol..

[14] Chen Y., Qian H., Zhu W., Zhang X., Yan Y., Ye S., Peng X., Li W., Xu W. (2010). Hepatocyte growth factor modification promotes the amelioration effects of human umbilical cord mesenchymal stem cells on rat acute kidney injury. Stem Cells Dev..

[15] Johansen T., Lamark T. (2011). Selective autophagy mediated by autophagic adapter proteins. Autophagy.

[16] De Rechter S., Decuypere J.-P., Ivanova E., van den Heuvel L.P., De Smedt H., Levtchenko E., Mekahli D. (2016). Autophagy in renal diseases. Pediatric Nephrol..

[17] Ding Y., Choi M.E. (2014). Autophagy in diabetic nephropathy. J. Endocrinol..

[18] Deng X., Xie Y., Zhang A. (2017). Advance of autophagy in chronic kidney diseases. Renal Fail..

[19] Zoncu R., Efeyan A., Sabatini D.M. (2011). mTOR: From growth signal integration to cancer, diabetes and ageing. Nat. Rev. Mol. Cell Biol..

[20] Lloberas N., Cruzado J.M., Franquesa M., Herrero-Fresneda I., Torras J., Alperovich G., Rama I., Vidal A., Grinyó J.M. (2006). Mammalian target of rapamycin pathway blockade slows progression of diabetic kidney disease in rats. J. Am. Soc. Nephrol..

[21] Mori H., Inoki K., Masutani K., Wakabayashi Y., Komai K., Nakagawa R., Guan K.-L., Yoshimura A. (2009). The mTOR pathway is highly activated in diabetic nephropathy and rapamycin has a strong therapeutic potential. Biochem. Biophys. Res. Commun..

[22] Gödel M., Hartleben B., Herbach N., Liu S., Zschiedrich S., Lu S., Debreczeni-Mór A., Lindenmeyer M.T., Rastaldi M.-P., Hartleben G. (2011). Role of mTOR in podocyte function and diabetic nephropathy in humans and mice. J. Clin. Invest..

[23] Bruno S., Grange C., Collino F., Deregibus M.C., Cantaluppi V., Biancone L., Tetta C., Camussi G. (2012). Microvesicles derived from mesenchymal stem cells enhance survival in a lethal model of acute kidney injury. PLoS ONE.

[24] Gatti S., Bruno S., Deregibus M.C., Sordi A., Cantaluppi V., Tetta C., Camussi G. (2011). Microvesicles derived from human adult mesenchymal stem cells protect against ischaemia–Reperfusion-induced acute and chronic kidney injury. Nephrol. Dialysis Transplantation.

[25] Yang J., Liu X.-X., Fan H., Tang Q., Shou Z.-X., Zuo D-.M., Zou Z., Xu M., Chen Q.-Y., Peng Y. (2015). Extracellular vesicles derived from bone marrow mesenchymal stem cells protect against experimental colitis via attenuating colon inflammation, oxidative stress and apoptosis. PLoS ONE.

[26] Lange-Consiglio A., Perrini C., Albini G., Modina S., Lodde V., Orsini E., Esposti P., Cremonesi F. (2017). Oviductal microvesicles and their effect on in vitro maturation of canine oocytes. Reproduction.

[27] Tesch G.H., Allen T.J. (2007). Rodent models of streptozotocin-induced diabetic nephropathy (Methods in Renal Research). Nephrology.

[28] Tang Y., Cai Q.-H., Wang Y.-J., Fan S.-H., Zhang Z.-F., Xiao M.-Q., Zhu J.-Y., Wu D.-M., Lu J., Zheng Y.-L. (2017). Protective effect of autophagy on endoplasmic reticulum stress-induced apoptosis of alveolar epithelial cells in rat models of COPD. Biosci. Rep..

[29] Hayat M.A. (1981). Principles and techniques of electron microscopy. Biological Applications.

[30] Yang B., El Nahas A.M., Thomas G.L., Haylor J.L., Watson P.F., Wagner B., Johnson T.S. (2001). Caspase-3 and apoptosis in experimental chronic renal scarring. Kidney Int..

[31] Wiame I., Remy S., Swennen R., Sagi L. (2000). Irreversible heat inactivation of DNase I without RNA degradation. BioTechniques.

[32] Helming L., Winter J., Gordon S. (2009). The scavenger receptor CD36 plays a role in cytokine-induced macrophage fusion. J. Cell Sci..

[33] Fleige S., Pfaffl M.W. (2006). RNA integrity and the effect on the real-time qRT-PCR performance. Mol. Aspects Med..

[34] Xu J.T., Zhao X., Yaster M., Tao Y.X. (2010). Expression and distribution of mTOR, p70S6K, 4E-BP1, and their phosphorylated counterparts in rat dorsal root ganglion and spinal cord dorsal horn. Brain Res..

[35] Wang X., Zhou G., Liu C., Wei R., Zhu S., Xu Y., Wu M., Miao Q. (2016). Acanthopanax versus 3-methyladenine ameliorates sodium taurocholate-induced severe acute pancreatitis by inhibiting the autophagic pathway in rats. Mediators Inflammation.

[36] Cao X., Wang Y., Wu C., Li X., Fu Z., Yang M., Bian W., Wang S., Song Y., Tang J. (2018). Cathelicidin-OA1, a novel antioxidant peptide identified from an amphibian, accelerates skin wound healing. Sci. Rep..

[37] Magalhaes J., Gegg M., Migdalska-Richards A., Schapira A. (2018). Effects of ambroxol on the autophagy-lysosome pathway and mitochondria in primary cortical neurons. Sci. Rep..

[38] Qian G., Liu D., Hu J., Gan F., Hou L., Zhai N., Chen X., Huang K. (2018). SeMet attenuates OTA-induced PCV2 replication promotion by inhibiting autophagy by activating the AKT/mTOR signaling pathway. Veterinary Res..

[39] Liu F., Bu Z., Zhao F., Xiao D. (2018). Increased T-helper 17 cell differentiation mediated by exosome-mediated micro RNA-451 redistribution in gastric cancer infiltrated T cells. Cancer Sci..

[40] Yuan J., Zhao X., Hu Y., Sun H., Gong G., Huang X., Chen X., Xia M., Sun C., Huang Q. (2018). Autophagy regulates the degeneration of the auditory cortex through the AMPK-mTOR-ULK1 signaling pathway. Int. J. Mol. Sci..

[41] Kitada M., Takeda A., Nagai T., Ito H., Kanasaki K., Koya D. (2011). Dietary restriction ameliorates diabetic nephropathy through anti-inflammatory effects and regulation of the autophagy via restoration of Sirt1 in diabetic Wistar fatty (*fa*/*fa*) rats: a model of type 2 diabetes. Exp. Diabetes Res..

[42] Thomas M.C., Cooper M.E., Zimmet P. (2016). Changing epidemiology of type 2 diabetes mellitus and associated chronic kidney disease. Nat. Rev. Nephrol..

[43] Mokdad A.H., Ford E.S., Bowman B.A., Dietz W.H., Vinicor F., Bales V.S., Marks J.S. (2003). Prevalence of obesity, diabetes, and obesity-related health risk factors, 2001. Jama.

[44] Tanaka Y., Kume S., Kitada M., Kanasaki K., Uzu T., Maegawa H., Koya D. (2011). Autophagy as a therapeutic target in diabetic nephropathy. Exp. Diabetes Res..

[45] Matboli M., Eissa S., Ibrahim D., Hegazy M.G., Imam S.S., Habib E.K. (2017). Caffeic acid attenuates diabetic kidney disease via modulation of autophagy in a high-fat diet/streptozotocin-induced diabetic rat. Sci. Rep..

[46] Abou-Hany H.O., Atef H., Said E., Elkashef H.A., Salem H.A. (2018). Crocin mediated amelioration of oxidative burden and inflammatory cascade suppresses diabetic nephropathy progression in diabetic rats. Chem. Biol. Interact..

[47] Duan P., Hu C., Quan C., Yu T., Huang W., Chen W., Tang S., Shi Y., Martin F.L., Yang K. (2017). 4-Nonylphenol induces autophagy and attenuates mTOR-p70S6K/4EBP1 signaling by modulating AMPK activation in Sertoli cells. Toxicol. Lett..

[48] Hsieh C.-Y., Miaw C.-L., Hsieh C.-C., Tseng H.-C., Yang Y.-H., Yen C.-H. (2009). Effects of chronic 4-n-nonylphenol treatment on aortic vasoconstriction and vasorelaxation in rats. Arch. Toxicol..

[49] Malik A., Mehmood M.H., Channa H., Akhtar M.S., Gilani A.-H. (2017). Pharmacological basis for the medicinal use of polyherbal formulation and its ingredients in cardiovascular disorders using rodents. BMC Complementary Altern. Med..

[50] Kriz W., LemLey K.V. (2017). Mechanical challenges to the glomerular filtration barrier: adaptations and pathway to sclerosis. Pediatric Nephrol..

[51] Swiatecka-Urban A. (2017). Endocytic trafficking at the mature podocyte slit diaphragm. Front. Pediatrics.

[52] Cara-Fuentes G., Clapp W.L., Johnson R.J., Garin E.H. (2016). Pathogenesis of proteinuria in idiopathic minimal change disease: Molecular mechanisms. Pediatric Nephrol..

[53] Lenoir O., Jasiek M., Hénique C., Guyonnet L., Hartleben B., Bork T., Chipont A., Flosseau K., Bensaada I., Schmitt A. (2015). Endothelial cell and podocyte autophagy synergistically protect from diabetes-induced glomerulosclerosis. Autophagy.

[54] Kang R., Zeh H., Lotze M., Tang D. (2011). The Beclin 1 network regulates autophagy and apoptosis. Cell Death Differ..

[55] Klionsky D.J., Abdelmohsen K., Abe A., Abedin M.J., Abeliovich H., Arozena A.A., Adachi H., Adams C.M., Adams P.D., Adeli K. (2016). Guidelines for the use and interpretation of assays for monitoring autophagy. Autophagy.

[56] Fang L., Zhou Y., Cao H., Wen P., Jiang L., He W., Dai C., Yang J. (2013). Autophagy attenuates diabetic glomerular damage through protection of hyperglycemia-induced podocyte injury. PLoS ONE.

[57] Liu W.J., Huang W.F., Ye L., Chen R.H., Yang C., Wu H.L., Pan Q.J., Liu H.F. (2018). The activity and role of autophagy in the pathogenesis of diabetic nephropathy. Eur. Rev. Med. Pharmacol. Sci..

[58] Lee C.-H., Inoki K., Guan K.-L. (2007). mTOR pathway as a target in tissue hypertrophy. Annu. Rev. Pharmacol. Toxicol..

[59] Chen J.-K., Chen J., Neilson E.G., Harris R.C. (2005). Role of mammalian target of rapamycin signaling in compensatory renal hypertrophy. J. Am. Soc. Nephrol..

[60] Yang Y., Wang J., Qin L., Shou Z., Zhao J., Wang H., Chen Y., Chen J. (2007). Rapamycin prevents early steps of the development of diabetic nephropathy in rats. Am. J. Nephrol..

[61] Inoki K., Mori H., Wang J., Suzuki T., Hong S., Yoshida S., Blattner S.M., Ikenoue T., Rüegg M.A., Hall M.N. (2011). mTORC1 activation in podocytes is a critical step in the development of diabetic nephropathy in mice. J. Clin. Invest..

[62] Gonzalez C.D., Lee M.-S., Marchetti P., Pietropaolo M., Towns R., Vaccaro M.I., Watada H., Wiley J.W. (2011). The emerging role of autophagy in the pathophysiology of diabetes mellitus. Autophagy.

[63] Yamahara K., Kume S., Koya D., Tanaka Y., Morita Y., Chin-Kanasaki M., Araki H., Isshiki K., Araki S.-I., Haneda M. (2013). Obesity-mediated autophagy insufficiency exacerbates proteinuria-induced tubulointerstitial lesions. J. Am. Soc. Nephrol..

[64] Peng K.Y., Horng L.Y., Sung H.C., Huang H.C., Wu R.T. (2011). Hepatocyte growth factor has a role in the amelioration of diabetic vascular complications via autophagic clearance of advanced glycation end products: Dispo85E, an HGF inducer, as a potential botanical drug. Metabolism.

[65] Hosokawa N., Hara T., Kaizuka T., Kishi C., Takamura A., Miura Y., Iemura S.-I., Natsume T., Takehana K., Yamada N. (2009). Nutrient-dependent mTORC1 association with the ULK1–Atg13–FIP200 complex required for autophagy. Mol. Biol. Cell.

[66] Mizushima N. (2010). The role of the Atg1/ULK1 complex in autophagy regulation. Curr. Opin. Cell Biol..

[67] Nagaishi K., Mizue Y., Chikenji T., Otani M., Nakano M., Konari N., Fujimiya M. (2016). Mesenchymal stem cell therapy ameliorates diabetic nephropathy via the paracrine effect of renal trophic factors including exosomes. Sci. Rep..

[68] Nassar W., El-Ansary M., Sabry D., Mostafa M.A., Fayad T., Kotb E., Temraz M., Saad A.N., Essa W., Adel H. (2016). Umbilical cord mesenchymal stem cells derived extracellular vesicles can safely ameliorate the progression of chronic kidney diseases. Biomater. Res..

[69] Komatsu M., Ichimura Y. (2010). Physiological significance of selective degradation of p62 by autophagy. FEBS Lett..

[70] Liu L., Jin X., Hu C.F., Li R., Zhou Z., Shen C.X. (2017). Exosomes derived from mesenchymal stem cells rescue myocardial ischaemia/reperfusion injury by inducing cardiomyocyte autophagy via AMPK and Akt pathways. Cell. Physiol. Biochem..

[71] Mu Y., Yan W.J., Yin T.L., Zhang Y., Li J., Yang J. (2017). Diet-induced obesity impairs spermatogenesis: A potential role for autophagy. Sci. Rep..

[72] Wu Y.T., Tan H.L., Shui G., Bauvy C., Huang Q., Wenk M.R., Ong C.N., Codogno P., Shen H.M. (2010). Dual role of 3-methyladenine in modulation of autophagy via different temporal patterns of inhibition on class I and III phosphoinositide 3-kinase. J. Biol. Chem..

[73] Verschooten L., Barrette K., Van Kelst S., Rubio Romero N., Proby C., De Vos R., Agostinis P., Garmyn M. (2012). Autophagy inhibitor chloroquine enhanced the cell death inducing effect of the flavonoid luteolin in metastatic squamous cell carcinoma cells. PLoS ONE.

